# Clinical and cost-effectiveness of contingency management for cannabis use in early psychosis: the CIRCLE randomised clinical trial

**DOI:** 10.1186/s12916-019-1395-5

**Published:** 2019-08-15

**Authors:** Luke Sheridan Rains, Louise Marston, Mark Hinton, Steven Marwaha, Thomas Craig, David Fowler, Michael King, Rumana Z. Omar, Paul McCrone, Jonathan Spencer, Joanne Taylor, Sophie Colman, Catherine Harder, Eleanor Gilbert, Amie Randhawa, Kirsty Labuschagne, Charlotte Jones, Theodora Stefanidou, Marina Christoforou, Meghan Craig, John Strang, Tim Weaver, Sonia Johnson

**Affiliations:** 1 Division of Psychiatry, University College London, London, UK; 2 Mental Health and Wellbeing, Warwick Medical School, University of Warwick, Coventry, UK; 3 Addictions Department, Institute of Psychiatry, Psychology, and Neuroscience, King’s College London, London, UK; 4 Health Service and Population Research Department, Institute of Psychiatry, Psychology and Neuroscience, King’s College London, London, UK; 5 Mental Health Social Work & Inter-professional Learning, Middlesex University London, London, UK; 6 Department of Health Services and Population Research, King’s Health Economics, Institute of Psychiatry, Psychology & Neuroscience, King’s College London, London, UK; 7 Department of Psychology, University of Sussex, Brighton, UK; 8 Department of Primary Care and Population Health and Priment Clinical Trials Unit, University College London, London, UK; 9 Department of Statistical Science, University College London, London, UK; 10 Institute for Mental Health, School of Psychology, University of Birmingham, Birmingham, B15 2TT UK; 11 Camden and Islington NHS Foundation Trust, 4 St Pancras Way, London, NW1 0PE UK; 12 Centre for Posttraumatic Mental Health, Department of Psychiatry, University of Melbourne, Melbourne, Australia

**Keywords:** Financial incentives, Contingency management, Cannabis, Psychosis, Early intervention, Substance misuse

## Abstract

**Background:**

Cannabis is the most commonly used illicit substance amongst people with psychosis. Continued cannabis use following the onset of psychosis is associated with poorer functional and clinical outcomes. However, finding effective ways of intervening has been very challenging. We examined the clinical and cost-effectiveness of adjunctive contingency management (CM), which involves incentives for abstinence from cannabis use, in people with a recent diagnosis of psychosis.

**Methods:**

CIRCLE was a pragmatic multi-centre randomised controlled trial. Participants were recruited via Early Intervention in Psychosis (EIP) services across the Midlands and South East of England. They had had at least one episode of clinically diagnosed psychosis (affective or non-affective); were aged 18 to 36; reported cannabis use in at least 12 out of the previous 24 weeks; and were not currently receiving treatment for cannabis misuse, or subject to a legal requirement for cannabis testing. Participants were randomised via a secure web-based service 1:1 to either an experimental arm, involving 12 weeks of CM plus a six-session psychoeducation package, or a control arm receiving the psychoeducation package only. The total potential voucher reward in the CM intervention was £240. The primary outcome was time to acute psychiatric care, operationalised as admission to an acute mental health service (including community alternatives to admission). Primary outcome data were collected from patient records at 18 months post-consent by assessors masked to allocation. The trial was registered with the ISRCTN registry, number ISRCTN33576045.

**Results:**

Five hundred fifty-one participants were recruited between June 2012 and April 2016. Primary outcome data were obtained for 272 (98%) in the CM (experimental) group and 259 (95%) in the control group. There was no statistically significant difference in time to acute psychiatric care (the primary outcome) (HR 1.03, 95% CI 0.76, 1.40) between groups. By 18 months, 90 (33%) of participants in the CM group, and 85 (30%) of the control groups had been admitted at least once to an acute psychiatric service. Amongst those who had experienced an acute psychiatric admission, the median time to admission was 196 days (IQR 82, 364) in the CM group and 245 days (IQR 99, 382) in the control group. Cost-effectiveness analyses suggest that there is an 81% likelihood that the intervention was cost-effective, mainly resulting from higher mean inpatient costs for the control group compared with the CM group; however, the cost difference between groups was not statistically significant. There were 58 adverse events, 27 in the CM group and 31 in the control group.

**Conclusions:**

Overall, these results suggest that CM is not an effective intervention for improving the time to acute psychiatric admission or reducing cannabis use in psychosis, at least at the level of voucher reward offered.

## Background

Cannabis is the most commonly used illicit substance amongst people with psychosis [1]. In longitudinal studies, cannabis use in first episode psychosis is associated with substantially higher acute psychiatric admission rates [2]: an Australian study reported a 51% admission rate over 15 months follow-up amongst substance users (mostly cannabis) compared with 17% amongst non-users [3], accompanied by a threefold difference in inpatient admission rates. Similarly, a Dutch study reported a 42% admission rate amongst persistent cannabis users compared with 17% amongst those who never used or stopped round the time of first onset [4]. A dose-response relationship between severity of cannabis misuse and time to admission was also reported in this study. Problematic cannabis use in psychosis is also associated with marked delays in remission [5], and lower engagement in work or education, as well as poorer outcomes in other clinical and social domains [3, 6, 7]. Despite the clear need for effective treatments in this group, so far none have been identified. A Cochrane review [8] of psychosocial interventions for substance misuse in severe mental illness identified 32 randomised controlled trials but found little evidence that any type of therapy was more effective than treatment as usual, including Cognitive Behavioural Therapy and Motivational Interviewing.

Contingency management (CM) is an operant conditioning-based intervention for substance misuse that typically uses financial rewards to reinforce target behaviours, such as abstinence from substance use or treatment adherence. CM has an increasingly substantial evidence base in a variety of contexts, including cannabis misuse [9–11], smoking cessation [12], heavy drinking [13], and other illicit drug misuse [14, 15]. The National Institute for Health and Care Excellence (NICE) advocates its adoption in England in the management of substance use [16]. In a review conducted by NICE as part of its guidance of psychosocial interventions for substance misuse, 14 trials of CM were identified, all from the USA, of which three involved cannabis use. A consistent finding of a benefit for CM was reported, with most studies using abstinence at 12 weeks as their outcome measure. In the context of severe mental illness, a number of trials have found CM to be effective in reducing use in patients with severe mental illness for alcohol [17], stimulant use [18], and cigarette smoking [19]. This includes one fairly recent trial of a 12-week CM intervention for stimulant misuse that found the treatment to be clinical and cost-effective [20].

However, to date there is little research on CM for cannabis use in severe mental illness, with only one randomised controlled trial [21] reported. The trial found that CM, combined with an enhanced treatment as usual psychosocial intervention (Supportive Treatment for Addiction Recovery), resulted in more drug-free urines, reduced hospitalisation, and better quality of life than treatment as usual alone. However, only a small proportion of participants abused cannabis (7%), with 93% abusing cocaine or heroin. Beyond this, there have also been three small feasibility studies [22–24], which taken together also provide some evidence that CM may be feasible, acceptable, and efficacious in this cohort. Furthermore, with the exception of a small number of recent evaluative studies in Europe [25], the evidence base for CM is drawn almost entirely from the USA. There is relatively little experience of using CM in the UK, with only a few studies reported in any clinical group. Some recent examples include the CONMAN trial, which provided an evidence base for CM in uptake of hepatitis B vaccines amongst opiate users [26], and FIAT, which found incentives to be effective for reinforcing adherence to antipsychotic medication [27].

The present study investigated the clinical and cost-effectiveness of CM for reducing cannabis use and thus time to acute psychiatric admission amongst Early Intervention in Psychosis (EIP) service users. The objectives were (1) to conduct a pilot study of a CM intervention for cannabis use in early psychosis, (2) if the pilot was successful, to proceed with a full multi-centre pragmatic randomised controlled trial, (3) to test whether the intervention results in an increase in the time to acute psychiatric admission (the primary outcome), (4) to test whether the intervention results in a decrease in cannabis use, reduced positive psychotic symptoms, and in an increase in participation in work or education (secondary outcomes), (5) to assess the cost-effectiveness of the intervention from an NHS perspective.

## Methods

### Trial design and participants

CIRCLE was a rater-blind, multi-centre randomised controlled trial with two arms. The experimental (CM) group received a 12-week CM intervention together with a Treatment-As-Usual (TAU) package targeting cannabis use, which was a standardised and optimised psychoeducational package of the type of psychoeducational intervention recommended in EIP practice [28]. The control group received the optimised TAU psychoeducational intervention only. Assessments were performed at the time of consent, at 3 months (treatment end), and at 18 months following trial inclusion. The primary outcome was time to acute psychiatric admission, operationalised as admission to an acute mental health service. Ethical approval for the trial was received on 16 March 2012 from the London – South East National Research Ethics Service committee (REC reference 11/LO/1939). The trial was conducted according to the trial protocol published by Johnson et al. [29], and trial procedures are described here only in brief. Oversight was provided by an external Trial Steering Group that was appointed as agreed by the funding body.

Participants were recruited via EIP services throughout the Midlands and South East of England. Sites were added to the trial until the recruitment target was achieved. EIP services accept individuals who have developed psychotic illness for the first time and aim to treat the initial episode early and effectively, minimising acute psychiatric admissions and optimising clinical and social recovery. They are now standard provision in English mental health Trusts following policy mandates, and typically work with service users for 3 years prior to discharge to primary care or to other mental health teams for continuing care [16]. Inclusion criteria were (1) on an EIP service caseload, (2) having used cannabis at least once in 12 out of the previous 24 weeks, (3) age 18–36, (4) living in stable accommodation, (5) sufficient English to understand fully and answer the assessment instruments, (6) able to give informed consent to participate, (7) not subject to a compulsory community treatment or court order requiring urine testing for cannabis, (8) not in receipt of treatment for cannabis use from another agency, and (9) not compulsorily detained in hospital, or in prison.

### Randomisation and masking

Following the baseline assessment interview, participants were randomised with a 1:1 ratio, stratified on severity of cannabis use (1–3 uses per week, > 3 uses per week). An independent randomisation service was used, coordinated by Priment Clinical Trials Unit based at University College London. Primary outcome assessors were masked to allocation throughout the trial. Secondary outcome assessors were masked to allocation at follow-up assessments in the main trial. However, masking was not feasible at the 3-month assessment interview in the pilot as sufficient researchers were not available for different staff to conduct initial and follow-up assessments. The statistical and health economics analysis plans were finalised before database lock and statisticians and health economists undertaking the analysis were masked to trial arm allocation.

### Procedures

The intervention under evaluation was 12 weeks of CM delivered by clinical staff, who included graduates without mental health professional qualifications, such as assistant psychologists. Participants attended weekly CM sessions at which they were immediately rewarded with vouchers if urinalysis indicated cannabis abstinence since the previous session. Urinalysis was performed using a small bench-top analyser capable of providing rapid test results of drug of misuse urinary concentration (Kaiwood CHR-110). To perform the analysis, the clinician pipetted a fixed amount of urine into a buffer solution tube to give a 7:1 serial dilution. This allowed a standard 50 ng/ml marijuana test cassette placed in the analyser to provide a urine cannabis concentration reading between 0 and 350 ng/ml. Guidelines were provided to all staff delivering the intervention to allow interpretation of the test results. The guidelines set out upper and lower thresholds for urinary tetrahydrocannabinol (THC) based on recommendations from the suppliers of the urinalysis equipment, Surescreen Diagnostics. Results greater than the upper threshold (350 ng/ml) clearly indicated use within the last week, so were not rewarded. Results below the lower threshold indicated urinary THC concentration below 50 ng/ml, the accepted standard for detecting urinary THC, so were rewarded. Within these thresholds, urinary THC was expected to fall each week until it reached the lower THC threshold, which should be reached within 1 month. A drop in urinary THC would receive the reward. A similar result to, or higher than, the previous week would fail. A temperature strip on the side of the specimen cup allowed staff to check whether the sample had been tampered with.

The CM schedule and rules were adapted from Budney et al. [10, 11], which both investigated CM for cannabis misuse. It used a variable reward schedule that began at £5 for providing a baseline sample in the first week. In the pilot, voucher values rose by £2 each week, with a £10 bonus voucher every other session. Based on feedback from clinicians and service users, the schedule was simplified in the main trial. The voucher value rose by £5 every two clean samples and the bonuses were removed. In total, participants could receive £240 (US$307, €267 equivalent (11 Jan. 2019)) in vouchers in both versions of the reward schedule. If a participant submitted a urine specimen indicating cannabis use in the previous week, they would receive a £5 voucher. Patients who failed to attend intervention sessions or submit a scheduled specimen did not receive a voucher. If the participant attended the next week and provided a negative sample, they were rewarded with £10. In the subsequent week, if the participant provided a second consecutive negative sample, the voucher values resumed from the highest previous level of reward. Participants could arrange in advance to miss scheduled sessions and still receive the reward for that week if they had a valid commitment that prevented them from attending and they subsequently demonstrated that they had not used since the previous sessions. They could do this on a maximum of two occasions for 1 week only each time. Vouchers were for major supermarkets.

The TAU was a standardised version of a good quality psychoeducation, which was delivered by EIP staff in a digital format. It was designed to be sufficiently highly structured for staff without high-level clinical qualifications, such as support workers or assistant psychologists, to be able to deliver it competently following brief training. The package was comprised of six modules that were delivered via a standard PC or laptop. Full delivery of all six modules was intended to take approximately 3 h, normally offered over six regularly programmed sessions of 30 min duration. It included a PDF package for clinicians to work through with their clients, which presents information regarding the effects of cannabis, motivational materials, and strategies for coping, minimising potential harms, and abstaining from cannabis. It also included video materials, short quizzes, audio files, and further information and written records of the modules for the service user to keep. The primary aim was to deliver information to meet psychoeducation goals that were tailored to the individual needs of the participant, but not to act as a psychological intervention. It adopted a harm minimisation approach, with an acknowledgement that in a young person with psychosis, cannabis abstinence may be required to ensure that no harm is done. The content was based on motivational interviewing principles, relapse prevention, and harm reduction strategies [link to psychoeducation package - https://www.ucl.ac.uk/psychiatry/research/epidemiology-and-applied-clinical-research-depa/projects/circle-trial/trial-materials]. A full day’s training to provide CM and the TAU psychoeducational package was delivered to EIP clinical staff by members of the CIRCLE research team. Training was tailored to the knowledge and experience of clinicians, some of whom were assistant psychologists with psychology degrees, but not mental health professional training. Clinicians were provided with written documentation on delivering the CM and the psychoeducation and supported by the CIRCLE team throughout the trial. Records of urinalysis results and voucher rewards received for each participant were checked by the CIRCLE research team to ensure fidelity to the intervention protocol.

### Outcomes

The primary outcome was time to admission to an acute psychiatric service, including psychiatric hospital, crisis resolution team or crisis house, or other acute mental health service intended as an equivalent to hospital. The primary outcome was assessed over an 18-month period using electronic patient records.

Secondary outcomes were between-group differences at follow-up for the following: (1) severity of positive symptoms of psychosis. (2) Social functioning based on self-reports of engagement in work or study. (3) Self-reported number of days’ cannabis use in the previous 3 months at 3-month follow-up, or the previous 6 months at 18 months. (4) Proportion of cannabis-free urines at assessment. (5) Number of admissions over 18 months follow-up. (6) Quality-adjusted life years (QALYs) (SF-12 and EQ-5D) and service use (CSRI) were used in the cost-effectiveness analyses. Baseline and secondary outcome data were collected primarily during assessment interviews. Some secondary outcome data were checked or collected using electronic patient records at each assessment. The following measures were performed at all assessment interviews:

- Demographics, most recent diagnosis, and social information. Where feasible, data were checked using patient records.
- Cannabis use
  - ○ The timeline followback (TLFB) [30] was used to record self-reported substance use, including cannabis, other illicit substances, and alcohol, over the previous 6 months at baseline and 18 months, and over the previous 3 months at the 3-month follow-up assessment interview.
  - ○ Structured Clinical Interview for DSM IV (SCID) part E was used to assess history of alcohol and substance misuse disorders.
  - ○ Urinalysis for cannabis, performed using a commercially available immunoassay test for metabolites of the cannabinoid tetrahydrocannabinol (THC) using the standard cut-off of 50 ng/ml [31].
- Psychotic symptoms
  - ○ The positive and negative symptom subscales of the Positive and Negative Syndrome Scale (PANSS) [32].
- Service use and health economic analysis:
  - ○ The Client Service Receipt Inventory (CSRI) [33] was used to record clinical service use, medication use, receipt of state welfare benefits, and use of other state-funded services including the criminal justice system. Data were collected for the previous 6 months at baseline and 18 months, and for the preceding 3 months at 3-month assessment. Data were collected at assessment interview and checked using patient records. To minimise attrition, at 18 months, data were collected from patient records for a subset of the resources deemed most likely to contribute to higher costs and that feasibly could be collected. Patient record data were collected for all patients still in the trial, unless they had withdrawn or their patient records could not be identified
  - ○ 12-Item Short Form Survey (SF-12) [34] and the EQ-5D [35] were used to derive quality-adjusted life years (QALYs).
  - ○ Details of the participant’s referral to the EIP service, history of admission to acute mental health services, and time spent on a Community Treatment Order (CTO) in the last 6 months at baseline. At 3 months and 18 months, history of admission to acute mental health services and time spent on CTO were recorded since study inclusion. Data were obtained from patient records

### Statistical methods

The sample size for the trial was 544. This was based on data suggesting a usual acute psychiatric admission rate of around 50% over the study timeframe in cannabis users [3, 4]. A 15% decrease in this rate due to the intervention was considered to be clinically beneficial. Using a power of 90% and a significance level of 5%, a total sample size of 460 subjects was determined to be required. This sample size was based on an analysis of time to acute psychiatric admission and allowed a 37% decrease in the hazard of admission (hazard ratio of 0.63) in the intervention group to be detected using a Cox proportional hazards model. The sample size was inflated by a factor of 1.06, which assumed that each clinician delivering the intervention would see an average of four trial participants, and an intraclass correlation coefficient of 0.02 for clinician clustering, which gave a total sample size of 488. Finally, the sample size was inflated by 10% to account for attrition for the primary outcome, giving a total sample size of 544. This calculation was performed using Stata version 11 [36].

All analyses were carried out comparing the CM and control groups as randomised using all available data. The continuous variables were summarised using mean (standard deviation (SD)) or median (interquartile range (IQR)) as appropriate. Categorical variables were presented as frequencies and percentages. Logistic regression was used to determine baseline predictors of missingness. Kaplan-Meier survival curves by randomised groups were used to examine the primary outcome (time to acute psychiatric admission) descriptively. A Cox proportional hazards model was used to compare the primary outcome in the intervention and control groups, adjusting for severity of cannabis use (the stratification variable; 1 to 3 times a week versus 4 or more times a week) at baseline and whether the participant was part of the pilot trial. The assumption of proportional hazards was checked using Schoenfeld residuals [37]. The supportive analyses for the primary outcome were as follows:

- Adjusting for significant baseline predictors of missingness.
- Excluding those participants who had no secondary outcome data (for 12 weeks and 18 months separately).
- Including those in the main trial only as some minor changes were made to the protocol at the end of the pilot before the main trial commenced
- Adjusting additionally for the number of psychoeducation sessions attended (which was offered in both arms of the trial).
- Adjusting additionally for the number of admissions in the 6 months prior to baseline.
- Adjusting for the same factors as the primary analysis but using centre as the clustering variable instead of clinician.

Secondary outcomes were analysed separately at 3 and 18 months. Models were adjusted for severity of cannabis use at baseline and whether the participant was part of the pilot trial. For the dichotomous outcomes (cannabis-positive urine, engaged in work or study), logistic regression was used. The residuals for the positive and negative symptoms from PANSS outcomes were not normally distributed and were therefore log transformed and analysed using linear regression models. Zero-inflated Poisson regression was used for count outcomes including number of cannabis days and number of admissions over follow-up, as there were excess zeros in these outcomes. The number of cannabis days at 3 months was analysed using Poisson regression. All secondary outcomes were also analysed after adjusting for predictors of missingness. An additional supportive analysis for number of admissions was to include all those who were discharged from psychiatric services within 18 months and assuming they did not have any admissions over 18 months. Robust standard errors were used in all regression models to account for clustering by a clinician delivering the CM in the analyses [38]. Results from all supportive and secondary analyses are presented as estimates and 95% confidence intervals (CI) as specified in the statistical analysis plan. All analyses were carried out using Stata version 14 [39].

### Health economic analyses

The cost-effectiveness analysis was conducted from an NHS/social care perspective over 18 months. Costs of the CM intervention and psychoeducation were calculated using the salaries of staff delivering the CM and psychoeducation, supervision time, and overheads. Other service use was measured with the CSRI and from data collected from patient records. Costs were calculated by combining resource use with 2015/2016 unit costs. Average costs were used to cover the gap between 3 and 12 months. This did not apply to inpatient costs which were available throughout the follow-up period. Imputation for missing non-inpatient costs was carried out with predictors being community costs for available periods and inpatient costs. (This was done so that analyses could be conducted on the sample with complete inpatient data.) Costs were compared between arms at 18-month follow-up using a regression model, controlling for baseline, and with bootstrapped confidence intervals based on the percentile method and 10,000 resamples.

Quality-adjusted life years (QALYs) were derived from the EQ-5D-3L and SF-12 (for secondary analyses) using area under the curve methods incorporating scores at baseline and 3- and 18-month follow-ups. Missing utility scores were imputed from all available scores. QALY comparisons were made after adjusting for baseline scores. Costs and QALY differences were obtained from 10,000 bootstrapped resamples. Cost-effectiveness planes and cost-effectiveness acceptability curves (CEACs) were generated.

## Results

### Recruitment and follow-up

**Fig. 1:**
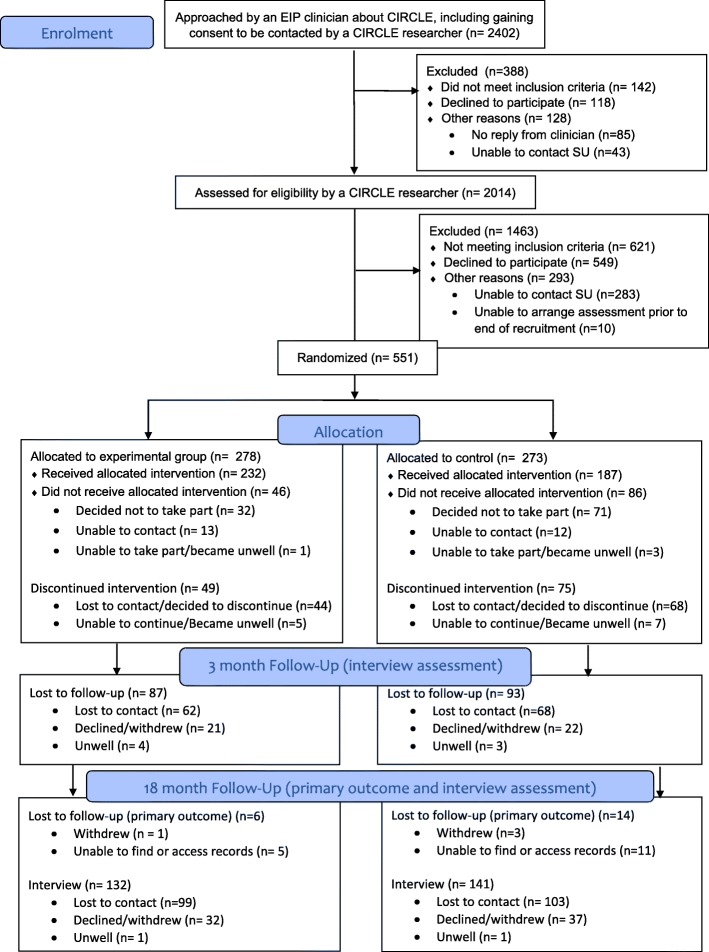
Consort diagram

Recruitment to the pilot phase occurred between June 2012 and March 2013. The pilot study successfully achieved its aims by recruiting within 10% of its target (*n* = 68), demonstrating the feasibility of recruiting, delivering the interventions, and retaining at least 60% of participants at 3 months. Subsequently, a full RCT was performed. Recruitment to the main trial occurred between October 2013 and March 2016. Follow-up data collection was completed by October 2017. Figure 1 shows the flow of service users through the trial. Seventy EIP teams took part in the trial from 23 NHS trusts. Two thousand four hundred two service users were initially approached by EIP clinicians. Of these, 551 service users gave consent and were randomised into the trial. Primary outcome data were obtained for 530 participants (92%). Assessment interviews were performed with 371 participants at 3-month follow-up (67%) and 278 participants at 18 months (50%).

### Baseline characteristics

Most baseline characteristics were similar between the two groups. In both groups, over 85% of participants were male, with a mean age of around 25 (SD 4). 72% were using cannabis more than three times a week and reported using on 111 days on average in the previous 6 months. Around a third of participants were diagnosed with schizophrenia or schizo-affective disorder and half had diagnoses of other types of psychosis, perhaps in part because EIP services tend to be reticent about making a diagnosis of such as schizophrenia because of concerns regarding stigma and diagnostic instability. A quarter of participants were currently engaged in some form of work or education, but a large majority (over 80%) had held open market employment at some point. Median PANSS positive symptom score was 12 (IQR 9, 17) in the control group and 13 (IQR 9, 16) in the CM group. Lifetime rates of cannabis dependence were very high (87% of control and 85% of intervention group members), and around three quarters in both groups were dependent at baseline. Lifetime rates of alcohol misuse or dependence, and of reports of using substances other than cannabis, were also fairly high (e.g. 47% of control and 52% of intervention group members reported using cocaine; 36% of control and 32% of intervention group members met the criteria for alcohol dependence) (Table 1). In the 6 months prior to baseline, median days of substance use other than cannabis were low (0) in the CM group (IQR 0, 4) and in the control group (0; IQR 0, 1). Median alcohol using days was 6 in both the CM (IQR 0, 26) and control groups (IQR 0, 26).

**Table 1:** Baseline demographics

Characteristic	Control		Contingency Management (CM)	
	*n*/*N* or median or utility score	%, SD, or IQR	*n*/*N* or median or utility score	%, SD, or IQR
Male	240/273	88	238/278	86
Age mean (SD)	25	(4)	24	(4)
Ethnicity				
White	144/273	53	148/277	53
Black	62/273	23	65/277	23
Asian	30/273	11	29/277	10
Other	37/273	14	35/277	13
Marital status				
Single	253/273	93	259/278	93
Married or cohabiting	14/273	5	17/278	6
Other	6/273	2	2/278	1
Educational attainment				
No qualifications	48/273	18	43/277	16
GCSE or equivalent	104/273	38	133/277	48
A level or equivalent	67/273	25	58/277	21
Post 18 education (including HND, trade, degree)	54/273	20	43/277	16
Ever had open market employment	223/273	82	240/278	86
Any current work or study	67/273	25	73/278	26
Diagnosis				
Schizophrenia or schizo-affective disorder	80/256	31	90/268	34
Bipolar affective disorder	26/256	10	19/268	7
Depression with psychotic symptoms	11/256	4	5/268	2
Other psychosis	139/256	54	154/268	57
Cannabis use				
1–3 times a week	77/273	28	78/278	28
More than 3 times a week	196/273	72	200/278	72
Any work or study	58/183	32	58/189	31
Cannabis-positive urine	210/262	80	214/272	79
Number of days using cannabis*	108	(67, 156)	114	(70, 162)
History of cannabis dependence	238/273	87	236/278	85
Current cannabis dependence	183/238	77	185/236	78
PANSS positive symptoms median (IQR)	12	(9, 17)	13	(9, 16)
PANSS negative symptoms median (IQR)	14	(11, 19)	14	(10, 19)
EQ-5D-3L utility score	0.7619		0.7652	
SF-6D utility score	0.6789		0.6833	

### Participation in CM and psychoeducation

Participants in the CM group obtained a mean of £64 (out of a maximum of £240) in voucher rewards and attended a median number of 9 (IQR 3, 12) (maximum of 12) CM sessions. Forty-six participants declined the CM intervention or did not attend any sessions. Participants attended a median of 6 and 4 (maximum of 6) psychoeducation sessions in the CM and control groups respectively. However, 86 participants in the control group and 63 in the CM group declined the psychoeducation or did not attend any sessions.

### Primary outcome

**Fig. 2:**
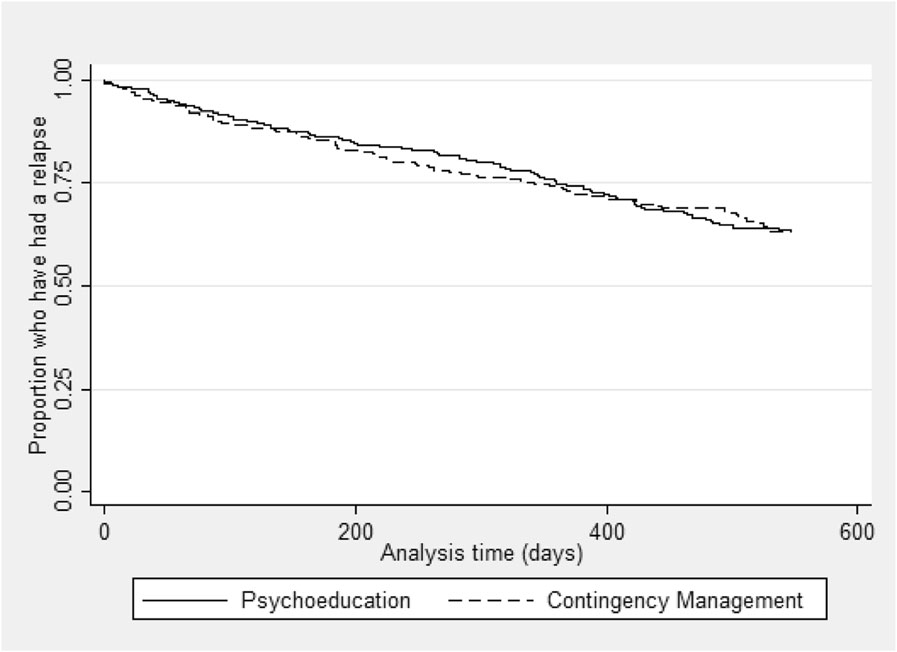
Kaplan-Meier survival curve by randomised group for the primary outcome, time to an acute psychiatric admission

For the primary outcome, there was no statistically significant difference in time to admission to an acute mental health service between the randomised groups (hazard ratio (HR) 1.03, 95% CI 0.76, 1.40) (Fig. 2 and Tables 2, 3, and 4).

**Table 2:** Outcomes at 3 months (treatment end) and 18 months

		Control		CM	
		*n*/*N*, mean, or median	% or (IQR)	*n*/*N*, mean, or median	% or (IQR)
Admitted to an acute mental health service	18 months	85/259	33	90/272	33
Number of admissions	18 months	0	(0, 1)	0	(0, 1)
Any work or study	3 months	58/183	32	58/189	31
	18 months	45/135	33	42/145	29
Cannabis-positive urine	3 months	122/170	72	128/184	70
	18 months	76/124	61	77/136	57
Number of days using cannabis* (median)	3 months	30	(3, 84)	26	(1, 67)
	18 months	26	(1, 142)	26	(0, 118)
PANSS positive symptoms (median)	3 months	11	(8, 16)	10	(8, 14)
	18 months	10	(8, 15)	11	(8, 13)
PANSS negative symptoms (median)	3 months	14	(10, 18)	12	(9, 17)
	18 months	12	(8, 17)	12	(9, 17)
Number of days of using illicit substances other than cannabis (median)	3 months	0	(0, 1)	0	(0, 1)
	18 months	0	(0, 2)	0	(0, 1)
Number of days using alcohol (median)	3 months	4	(0, 12)	4	(0, 15)
	18 months	6	(0, 24)	6	(0, 24)
EQ-5D-3L utility score (mean)	3 months	0.7729		0.8175	
	18 months	0.8032		0.8336	
SF-6D utility score (mean)	3 months	0.7018		0.7114	
	18 months	0.7110		0.7205	
Number of psychoeducation sessions attended (median)		4	(0, 6)	6	(1, 6)
Number of contingency management sessions attended (median)				9	(3, 12)
Number who attended 4 or more PE sessions (used in the post hoc analysis)		137/263	52	168/273	62

*At 3 months, this was for the previous 12 weeks/84 days. At 18 months, this was for the previous 168 days

**Table 3:** Analysis of primary and secondary outcomes in terms of contingency management

Outcome	Estimate*	95% CI	*N*	Estimate†	95% CI	N
Time to acute psychiatric admission (HR)	1.03	(0.76, 1.40)	531	1.02	(0.75, 1.40)	531
Cannabis-positive urine sample 3 months (OR)	0.86	(0.56, 1.34)	354	0.85	(0.55, 1.32)	352
Cannabis-positive urine sample 18 months (OR)	0.84	(0.49, 1.41)	260	0.85	(0.50, 1.43)	260
log PANSS positive symptoms 3 months	− 0.07	(− 0.14, − 0.00)	366	− 0.07	(− 0.14, − 0.00)	364
log PANSS positive symptoms 18 months	−0.04	(− 0.13, 0.05)	276	− 0.04	(− 0.13, 0.04)	276
log PANSS negative symptoms 3 months	− 0.08	(− 0.16, 0.00)	362	− 0.08	(− 0.16, 0.00)	360
log PANSS negative symptoms 18 months	0.01	(− 0.08, 0.11)	276	0.02	(− 0.08, 0.11)	276
Paid work or study at 3 months (OR)	0.95	(0.62, 1.46)	372	0.94	(0.60, 1.47)	370
Paid work or study at 18 months (OR)	0.82	(0.50, 1.35)	280	0.82	(0.50, 1.35)	280
Number of days cannabis used in the previous 12 weeks (3 months follow-up) (IRR)	0.89	(0.75, 1.04)	371	0.88	(0.75, 1.04)	369
Number of days cannabis used in the previous 6 months (18 months follow-up) (IRR)	1.09	(0.88, 1.36)	274	1.08	(0.87, 1.33)	274
Number of admissions over 18 months follow-up	1.08	(0.75, 1.54)	374	1.09	(0.76, 1.55)	374
At least one admission over 18 months follow-up (OR)	1.02	(0.70, 1.48)	531	1.01	(0.69, 1.48)	531

*CI* confidence interval, *HR* hazard ratio, *OR* odds ratio, *IRR* incident rate ratio

* Adjusting for level of cannabis use at baseline and whether in the pilot study

† Additionally adjusting for baseline predictors of missingness. These are:

Time to admission, number of admissions, at least one admission: any work or study

Urine positive 3 months, PANSS positive 3 months, PANSS negative 3 months, any work or study 3 months, number of days cannabis use 3 months: exempt from work due to disability

Urine positive 18 months: any work or study

PANSS positive 18 months, PANSS negative 18 months, any work or study 18 months, number of days cannabis use 18 months: voluntary work

**Table 4:** Service use for the health economics

Service	Control			CM		
	% users	Mean (SD contacts)	Mean (SD) cost	% users	Mean (SD) contacts	Mean (SD) Cost
Inpatient stays	25.5	90.9	11,931	24.6	89.4	11,339
Early intervention team	68.9	11.1	303	68.7	11.7	302
GP	44.7	2.9	134	51.1	3.1	127
Psychiatrist	53.1	3.2	479	54.3	3.2	404
Psychologist	20.9	5.6	445	22.7	5.0	416
Home treatment/crisis team	10.3	12.8	21	9.0	10.7	302
Mental health nurse	13.9	7.0	129	15.1	9.1	127
Adult education class	3.7	9.0	18	2.5	5.4	404
Assertive outreach team	1.1	9.0	5	1.8	8.4	4
Class/group at a leisure centre	4.4	25.2	33	4.0	14.6	10
Community mental health centre	2.6	7.6	40	4.3	8.8	79
Day care centre/day hospital	0.7	27.0	3	1.4	5.2	4
Drop-in centre	4.8	10.8	33	1.4	16.5	12
Drug/alcohol service	4.8	6	23	4.0	16.0	163
Drug and alcohol advisor	10.6	7.9	116	9.4	5.5	291
Occupational therapist	7.0	3.7	62	6.1	2.1	37
Other counsellor/therapist	5.1	7.6	156	4.0	7.6	53
Other doctor	8.1	3.7	75	7.6	2.1	26
Self-help/support group	5.1	6.5	9	7.2	6.8	16
Social worker	10.3	7.5	58	7.9	10.4	44
Medication			288			308

### Supportive analyses

Results from the supportive analyses were similar. The odds of at least one admission over 18 months of follow-up were similar across randomised groups (OR 1.02, 95% CI 0.70, 1.48). Approximately, a third of participants experienced at least one acute admission to a mental health service, including hospital alternatives such as crisis teams and crisis houses, by 18 months in both the CM (90/272) and control groups (85/259). Amongst those who experienced an acute psychiatric admission, the median number of days until admission was 245 (IQR 99, 382) in the control group and 196 (IQR 82, 364) in the CM group.

### Secondary outcomes

For participants who had a full 18 months’ follow-up, those in the CM group had a slightly higher rate ratio for number of admissions than those in the control group (incidence rate ratio (IRR) 1.08, 95% CI 0.75, 1.54); this changed little when assuming those who were discharged from services had no admissions during follow-up or when including predictors of missingness. Those in the CM group had lower odds of paid work or study at both 3 and 18 months (OR 0.95, 95% CI 0.62, 1.46; OR 0.82, 95% CI 0.50, 1.35 respectively). However, those in the CM group also had slightly lower odds of cannabis-positive urine at 3 and 18 months (OR 0.86, 95% CI 0.56, 1.34; OR 0.84, 95% CI 0.49, 1.41 respectively). None of these results approached statistical significance at the 5% level. Illicit substance use other than cannabis was very low at both follow-ups (3-month median days of use: 0; IQR 0, 1 in both groups; 18-month: 0 in both groups, IQR 0, 2 in control; IQR 0, 1 in CM). The median number of alcohol-using days was 4 (control IQR 0, 12; CM IQR 0, 15) in both groups at 3 months and 6 (IQR 0, 24) in both groups in 18 months. For the log-transformed PANSS positive outcome at 3 months, the CM group score was on average 7% lower (better) than the control group (95% CI − 14%, − 0%). There were 58 reported serious adverse events (SAE). In the CM group, these were 24 psychiatric hospital admissions and 3 deaths. In the control group, there were 28 psychiatric hospital episodes, 2 deaths, and 1 arrest.^1^

### CM in the context of psychoeducation

A post hoc analysis was performed to help understand the results in the context of whether people had received psychoeducation as planned. Attending at least four of the six psychoeducation sessions planned was selected as the measure of compliance (137/263 (52%) in the control group and 168/273 (62%) in the CM group). There were no marked differences in demographic, social, or clinical baseline characteristics between compliers and non-compliers between randomised groups. A dichotomous variable indicating compliance was created. A Cox model with robust standard errors was conducted. The primary outcome of the trial (time to acute psychiatric admission) was used as the dependent variable in the analysis. Randomisation group, compliance, severity of cannabis use at baseline, and whether the participant was part of the pilot trial were included as covariates, and an interaction term between compliance and randomisation groups was used. The interaction term was statistically significant (*p* value for interaction 0.016), which suggests that CM might be effective in participants who receive sufficient psychoeducation. This may be due to the psychoeducation being effective amongst those who engaged with treatment, and engagement was higher in the CM group.

### Health economics

Intervention costs were on average £298 for the CM group and £140 for controls. Inpatient use (based on 531 participants) over the follow-up period was virtually the same between trial arms, with around one quarter receiving this and for about 3 months across the period (Table 4). Other service use (based on the smaller sample of 231 who were followed up with the CSRI) was relatively similar between the arms over the follow-up period. Costs for drug and alcohol services were however greater for the CM group. After imputation, the mean (SD) health and social care costs over the 18-month period were £15,614 (£29,360) for the CM group and £16,620 (£33,283) for controls. After adjusting for baseline costs, the CM group had costs that were on average £1625 lower than for controls (bootstrapped 95% CI, − £3355 to £6869).

**Fig. 3:**
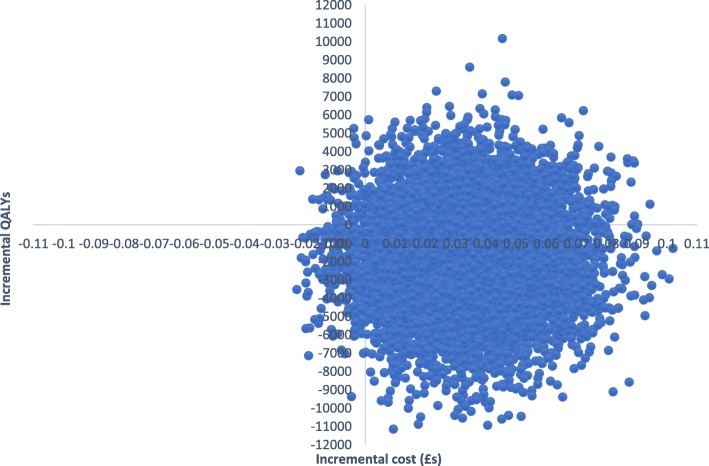
Cost-effectiveness plane based on EQ-5D-3L

**Fig. 4:**
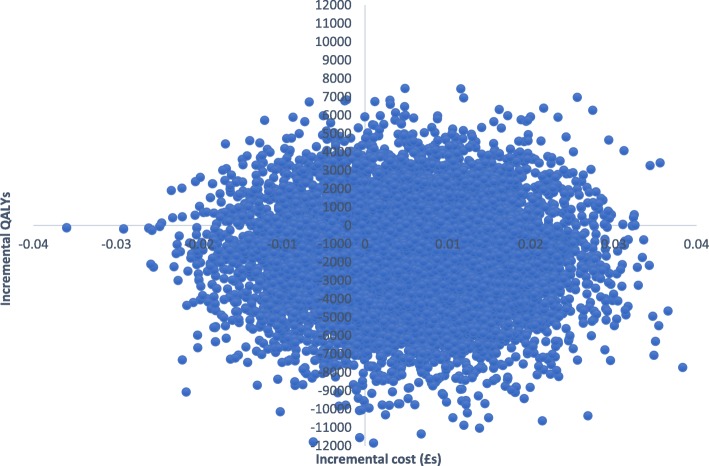
Cost-effectiveness plane based on SF-6D

**Fig. 5:**
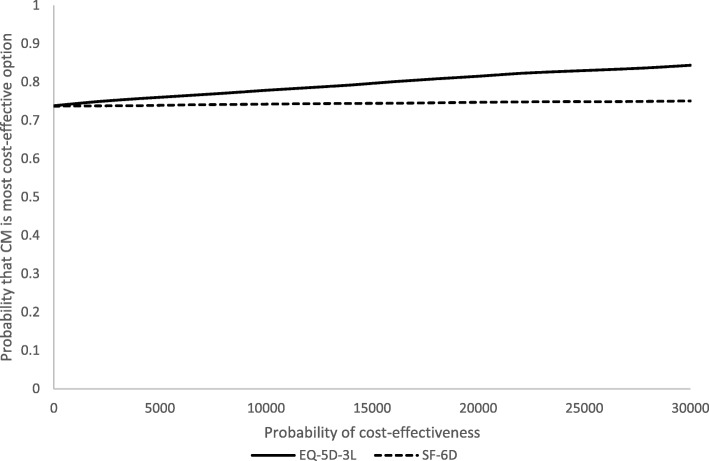
Cost-effectiveness acceptability curves

EQ-5D-3L and SF-6D utility scores increased gradually over time for both groups (Table 2). The total mean QALYs over the follow-up based on the EQ-5D-5L were 1.2218 for the CM group and 1.1855 for controls. Adjusting for baseline utility, CM resulted in 0.034 more QALYs than controls. QALYs based on the SF-6D scores were 1.0682 for CM and 1.0585 for controls. With adjustment, CM resulted in 0.0063 more QALYs than controls. Therefore, CM had lower costs and produced more QALYs and so ‘dominated’ the control. There is uncertainty around the results shown by the cost-effectiveness planes. With the EQ-5D-3L (Fig. 3), the most likely result is that CM has lower costs and better outcomes (72% of replications), followed by higher costs and better outcomes (26%), lower costs and worse outcomes (2%), and higher costs and worse outcomes (1%). With the SF-6D, there is more uncertainty (Fig. 4), but the most likely outcome is still CM having lower costs and better outcomes (52%), followed by lower costs and worse outcomes (22%), higher costs and better outcomes (18%), and higher costs and worse outcomes (8%). The CEACs (Fig. 5) show that even with a zero value attached to a QALY there is still a high probability of CM being the most cost-effective option. At a threshold value of £20,000, the probabilities are 0.81 for the EQ-5D-3L and 0.75 for the SF-6D.

## Discussion

The results of this trial indicate that CM confers no clinical advantage over TAU for patients with psychosis who use cannabis. Neither was any effect seen in our secondary outcomes, including cannabis use, engagement in work or education, and positive psychotic symptoms. However, a post hoc analysis found that compliance with psychoeducation in the CM arm resulted in a statistically significant improvement in time to acute psychiatric admission, while the same was not true for the control group. This suggests that CM had a clinical benefit amongst those who also engaged with psychoeducation.

The economic analyses show that the costs associated with CM were less than for the TAU, although the difference was not statistically significant and this analysis was carried out after imputation for a large proportion of cases, while we had primary outcome data for most who entered the trial. QALY differences based on the EQ-5D-3L were relatively large compared to other studies, but less so with the SF-6D. The lower costs and greater number of QALYs mean that CM is more cost-effective than TAU even though the differences for both costs and QALYs were not statistically significant. While interpretation of this is complex as there was little difference between CM groups in the intention-to-treat analyses, one possible explanation is that time to acute psychiatric admission was better amongst those who complied with psychoeducation in the CM group, but not the control group. The admission rate was lower than anticipated in the trial as a whole. Only around a third in each group required admission to acute care, while it was expected [3, 4] for the purposes of our sample size calculation and based on previous literature that half would experience an acute psychiatric admission. This suggests that a comparatively stable group of patients was recruited to trial and/or that the psychoeducation and/or the extra attention of being in a research study had a beneficial impact. Although the majority still had cannabis-positive urine, the number of reported cannabis-using days in the previous 6 months fell from over 100 to 26 in each group by 18-month follow-up, indicating that cannabis use declined in both groups over this period. This could be due to the psychoeducation intervention, the normal progression of use in this group, regression to the mean, or a combination of these factors. Low levels of self-reported illicit substance and alcohol use at follow-up indicate that people were not substituting them for cannabis.

Overall, the results of the trial are mixed, and stand in contrast to the frequently positive benefits reported for CM interventions [15, 16], including for substance misuse in psychosis [17–19, 21]. Reasons for this are unclear; however, some possible explanations include the following: Firstly, offering more frequent CM sessions or offering a higher reward might have been more clinically effective. The CM schedule was adapted from two trials by Budney et al. [10, 11], which both found a positive effect in cannabis misuse. However, the reward sessions in one of those trials occurred once per week, while in the other they occurred twice per week. Bellack et al. [21], which is the only other trial of CM to include cannabis in severe mental illness, also used a twice weekly reward schedule. CIRCLE was intended to be a pragmatic trial of a CM intervention in an EIP context, and based on feedback from EIP clinicians, it was thought that delivering sessions more frequently than once per week would not be feasible, while the reward value was intended to be substantial enough to incentivise abstinence without being viewed as too lavish and thus ethically problematic, and was supported by clinicians, service users involved in the patient and public involvement consultation, and experts in the field. It was also approximately in line with other trials in this field (e.g. [10, 21]). However, it was lower than that offered in some feasibility studies that found a positive effect (e.g. [22, 23]). Further discussions with stakeholders and experts in the field might elucidate this issue.

Secondly, cannabis dependence was high in this sample (around three quarters of participants at baseline), and those with dependence may find it harder to change their behaviour compared to those with less-severe problematic cannabis use. However, in the two trials by Budney et al. [10, 11] and Bellack et al. [21], all participants were dependent, and so it is unlikely that the CM intervention failed to provide a benefit because of high rates of dependence. An alternative explanation is that participants may have been using more highly potent forms of the cannabis and consequently may have found it more difficult to abstain than those using less-potent forms. There is good evidence that this cohort typically uses more potent forms of cannabis than non-psychiatric groups [40] and that high potency cannabis use is relatively prevalent in London, where much of our sample was recruited [41]. It is therefore plausible that use of highly potent types of cannabis may have been relatively widespread in our sample. However, we did not systematically record the type of cannabis participants were using, making further exploration of this possibility difficult.

Thirdly, while engagement was good with people attending a median of 4 psychoeducation sessions in the control group and 6 in the CM group, a substantial proportion of people (around one third of people in the control group and one fifth in the CM group) declined to take part in either the CM and/or the PE or did not attend any sessions. This suggests there may have been a substantial minority who were not particularly motivated to quit cannabis, but perhaps participated due to the enthusiastic approach to recruitment by staff and researchers and/or the small payment received to acknowledge baseline interview assessment participation. Recruitment was slower than anticipated, partly because fewer service users than expected wanted to enter treatment for cannabis use. The post hoc analysis results suggested that people who were motivated to adhere to the psychoeducation had better outcomes. It may be that a more engaged cohort would have benefited more from the CM.

Fourthly, patients in this cohort are often multiply disadvantaged [2, 5, 42], which is likely to make behavioural change much more challenging. This includes being less likely to be in work or education, having poorer social networks, and thereby being more socially isolated but also the nature of psychotic illness itself, which can include greater disorganisation, poorer social skills, and lower motivation [43, 44]. It may be that interventions are needed in this cohort that have a broader focus than just reducing cannabis use. It may be that a better approach would support patients in becoming less socially isolated or spending less time with cannabis-using peers, as well as helping them back into work or engaging in other meaningful activities.

Finally, the trial focused only on cannabis, but baseline data indicated considerable history of alcohol and substance use disorders. It may be that the CM would have been more effective if it had addressed these additional substances as well.

There were several limitations to the study. Firstly, CIRCLE was a pragmatic trial of a CM intervention delivered in EIP services in the UK. The CM and psychoeducation treatments were designed to be feasible for EIP clinicians to deliver in routine practice, which required limiting the additional workload to clinicians. As such, during the intervention period, urinalysis samples were only collected from the CM group and not controls. This makes it more challenging to analyse whether the CM group participants reduced their use more than the controls during the intervention period. However, self-reported days of cannabis use at treatment end did not differ between groups, suggesting no impact from the CM. However, CIRCLE should be viewed as a trial of a pragmatic CM intervention rather as a definitive trial of whether CM reduces cannabis use in psychosis. Studies that more rigorously assess drug use in both treatment groups are needed to investigate whether CM could be effective at reducing use in psychosis.

Secondly, the inclusion of an active control makes it more difficult to interpret results. The fall in cannabis use across the trial population suggests there may have been benefit from the enhanced TAU. While it was intended to be a standardised form of treatment as usual, it became apparent that it was a much better developed and more ambitious psychoeducation than was otherwise available in many of the participating EIP teams.

Thirdly, originally, the CM (and psychoeducation) was originally intended to be delivered by care coordinators (the nurses, occupational therapist or social workers with primary responsibility for keeping in touch with patients and organising their care). However, few care coordinators were prepared to deliver the interventions, largely due to concerns regarding time pressures and potential disruption to therapeutic relationships. Instead, other clinical staff, such as support workers or assistant psychologists, were trained to deliver it. Greater integration into routine care may have improved its effectiveness. However, data on delivery of the CM intervention suggested that those delivering it did adhere well to the intended protocols, and it may be that care coordinators would have been less successful in this.

Fourthly, while the follow-up rate for the primary outcome was very high, attrition was greater than anticipated on the interview measures, potentially introducing response bias.

Fifthly, while a standard threshold was used for the urinalysis at assessment interview (50 ng/ml), a lower threshold would have given a slightly more accurate measure of abstinence rates. A lower threshold may have identified a small difference between groups. However, as there was no difference in either the proportion of cannabis-free urine using a threshold of 50 ng/ml or self-reported days of use at either follow-up, it seems unlikely that a clinically significant difference would be identified.

Sixthly, while we approached all EIP patients who were identified to trial researchers as potentially eligible by their clinicians and who agreed to be contacted by a trial researcher, it may be that gate-keeping by EIP clinicians or self-selection by patients could have introduced bias. However, this was a pragmatic RCT and it is likely that the trial sample is a good reflection of the range of characteristics of the EIP service users who would enter treatment if the intervention was offered through EIP or specialist drug treatment services. Similar issues seem likely if the intervention was introduced as part of routine care.

Seventhly, we did not systematically record the type of cannabis participants were using. It may be that those using more potent types of cannabis would respond differently to the intervention compared to those using milder forms, either by finding it harder to change behaviour, or potentially by showing greater benefit from the CM treatment in terms of acute psychiatric service use.

Eighthly, although not a limitation in the analyses, the finding that CM has over 80% likelihood of being cost-effective needs to be treated with caution. In the sample that were followed up at both time points, the costs were lower and QALY outcomes better for the CM group compared to controls, and this led to a favourable cost-effectiveness ratio for CM which applied to most bootstrapped resamples. The cost and QALY differences were though limited and not statistically significant.

Finally, using acute psychiatric admission as the outcome is a pragmatic choice because it is routinely available data that are relatively accessible via patient records. But it is limited in that some acute psychiatric episodes are likely to be contained without acute service use, for example, it may be handled by the EIP team instead, and also that thresholds for admission to acute care may vary considerably, for example by clinician and by area.

## Conclusions

Overall, the lack of effectiveness of CM in these intention-to-treat analyses means that it cannot be recommended as an intervention to reduce cannabis use in patients with recent onset psychosis. However, the adherence and health economic analyses suggest that effectiveness of CM might be better amongst those who engage well with psychoeducation, or if a different reward value or schedule was offered. Further investigation of stakeholder perspectives would be useful to explore the latter possibility. However, overall, this is another trial that demonstrates how challenging it is to address the problem of cannabis use in psychosis. It may be that a substantially different approach is required to address this significant clinical problem. It has been noted that young people who have psychosis and problematic cannabis use are often multiply disadvantaged. Despite this, trials of interventions in this area are often narrowly focused on changing cannabis use. A more inclusive management that takes in patients’ social contexts, including engagement in work or education, might prove more fruitful.

## Data Availability

The datasets used and/or analysed during the current study are available from the corresponding author on reasonable request.

## Abbreviations

CEACs: Cost-effectiveness acceptability curves
CI: Confidence intervals
CM: Contingency management
CSRI: Client Service Receipt Inventory
CTO: Community Treatment Order
EIP: Early Intervention in Psychosis
HR: Hazard ratio
IQR: Interquartile range
NICE: National Institute for Health and Care Excellence
PANSS: Positive and Negative Syndrome Scale
QALYs: Quality-adjusted life years
SCID: Structured Clinical Interview for DSM IV
SD: Standard deviation
SF-12: 12-Item Short Form Survey
TAU: Treatment-As-Usual
THC: Tetrahydrocannabinol
TLFB: Timeline followback

## Footnotes

1 While all SAEs were recorded during the intervention period, as the research team did not have regular, direct contact with participants during the post-treatment follow-up phase, detection of SAEs was heavily dependent on EIP clinicians reporting them to the research team. While all deaths were reported by the EIP teams and recorded as SAEs, primary data collection shows that not all arrests or inpatient episodes were reported to the research team once the intervention had ended
